# Kallikrein-Related Peptidase 6 (KLK6) as a Contributor toward an Aggressive Cancer Cell Phenotype: A Potential Role in Colon Cancer Peritoneal Metastasis

**DOI:** 10.3390/biom12071003

**Published:** 2022-07-19

**Authors:** Hayet Bouzid, Feryel Soualmia, Katerina Oikonomopoulou, Antoninus Soosaipillai, Francine Walker, Khaoula Louati, Rea Lo Dico, Marc Pocard, Chahrazade El Amri, Natalia A. Ignatenko, Dalila Darmoul

**Affiliations:** 1 Institut National de la Santé et de la Recherche Médicale (INSERM) INSERM-1275, Hôpital Lariboisière, 75010 Paris, France; hayet.bouzid@cri-paris.org (H.B.); khawla.louati27@gmail.com (K.L.); rea.lo-dico@aphp.fr (R.L.D.); marc.pocard@inserm.fr (M.P.); 2 Université de Paris Cité, 75010 Paris, France; 3 Sorbonne Université, IBPS, UMR 8256 CNRS-UPMC, ERL INSERM U1164, Biological Adaptation and Ageing, 75005 Paris, France; feryel.soualmia@gmail.com (F.S.); chahrazade.el_amri@sorbonne-universite.fr (C.E.A.); 4 Schroeder Arthritis Institute, Krembil Research Institute, University Health Network, Toronto, ON M5T 3L9, Canada; katerina.oikonomopoulou@uhnresearch.ca; 5 Department of Pathology and Laboratory Medicine, Mount Sinai Hospital, Toronto, ON M5T 3L9, Canada; antoninus.soosaipillai@sinaihealth.ca; 6 Department of Pathology, Hôpital Bichat-Claude Bernard, 75018 Paris, France; francinecombrouze@gmail.com; 7 University of Arizona Cancer Center, University of Arizona, Tucson, AZ 85724, USA; nai@arizona.edu

**Keywords:** kallikrein-related peptidase 6, protease-activated receptors, colorectal cancer, metastasis, signaling, extracellular matrix

## Abstract

Kallikrein-related peptidases (KLKs) are implicated in many cancer-related processes. KLK6, one of the 15 KLK family members, is a promising biomarker for diagnosis of many cancers and has been associated with poor prognosis of colorectal cancer (CRC) patients. Herein, we evaluated the expression and cellular functions of KLK6 in colon cancer-derived cell lines and in clinical samples from CRC patients. We showed that, although many KLKs transcripts are upregulated in colon cancer-derived cell lines, KLK6, KLK10, and KLK11 are the most highly secreted proteins. KLK6 induced calcium flux in HT29 cells by activation and internalization of protease-activated receptor 2 (PAR2). Furthermore, KLK6 induced extracellular signal–regulated kinases 1 and 2 (ERK1/2) phosphorylation. KLK6 suppression in HCT-116 colon cancer cells decreased the colony formation, increased cell adhesion to extracellular matrix proteins, and reduced spheroid formation and compaction. Immunohistochemistry (IHC) analysis demonstrated ectopic expression of KLK6 in human colon adenocarcinomas but not in normal epithelia. Importantly, high levels of KLK6 protein were detected in the ascites of CRC patients with peritoneal metastasis, but not in benign ascites. These data indicate that KLK6 overexpression is associated with aggressive CRC, and may be applied to differentiate between benign and malignant ascites.

## 1. Introduction

Tumor cell invasion and metastasis are major obstacles to successful treatment of colorectal carcinoma (CRC) patients. Most frequently, distant metastases from CRC are those to the liver and the peritoneum, i.e., peritoneal metastases (PM). Peritoneal spread of tumor cells is associated with an extremely poor prognosis [1]. Much progress has been made in overall understanding of tumor biology and metastasis [2], but knowledge on the factors influencing the metastatic route of tumor cells—especially in colorectal cancer—remains poor. Several hallmarks of aggressive cancers are a direct result of proteolytic activity, including tumor cell invasion into the stroma, angiogenesis, and metastasis [3]. Interestingly, a recent study demonstrated that the level of certain serine proteases in serum and tissue interstitial fluids can serve as an indicator of CRC progression [4]. Secreted members of the serine protease family turned up as key signaling molecules involved in tumor progression by activating particular targets including growth factors and members of a subfamily of four cell surface G-protein coupled receptors, termed ‘protease-activated receptors’ (PARs 1–4). Originally, PAR1, PAR3, and PAR4 are activated by thrombin, whereas PAR2 is activated by trypsin and also other trypsin-like serine proteases, but not by thrombin [5]. PARs are activated by N-terminal proteolytic cleavage. Upon removal of specific N-terminal peptides, the resulting N-termini serve as tethered activation ligand. Short synthetic peptides (activating peptides or APs) corresponding to the newly exposed amino-terminus are able to activate a given PAR receptor selectively and mimic cellular effects of the protease in the absence of proteolytic cleavage [5]. Once activated, PARs trigger a cascade of signal transduction pathways including the hydrolysis of phosphoinositide to yield inositol tris-phosphate and diacyl-glycerol, with the subsequent activation of protein kinase C and an elevation of intracellular calcium [5]. We have shown that thrombin and trypsin signal through PAR1 and PAR2 to stimulate cell proliferation and motility in human colon cancer cells through transactivation of receptor tyrosine kinases and MAP-kinases signaling [6,7,8,9]. More recently, we have shown that kallikrein-related peptidases (KLKs) trigger intracellular signaling pathways by activating protease-activated receptor (PAR) activators [10,11].

The kallikrein-related peptidases (KLK1–KLK15) are a family of trypsin- and chymotrypsin-like serine proteases, which are secreted into the extracellular space of many tissues. They are deregulated in neoplasia (mainly in adenocarcinomas) [10,12,13], and their upregulation is often associated with poor patient prognosis [10,11]. Accumulating evidence demonstrates that many kallikreins are involved in many cancer-related processes by controlling cell-growth, angiogenesis, invasion, and metastasis [10]. KLKs can affect cell function by producing bioactive peptides from their precursor, by activating proteolytic processes and by cleaving cell adhesion molecules [12,13]. We have shown that certain members of the cancer-associated kallikrein-related peptidases signal via PARs to control colon cancer tumorigenesis [14,15,16].

Elevated expression of KLK6 is a common feature in many human malignancies, including colon cancer progression (reviewed in [17]). Numerous studies have evaluated KLK6 as a promising biomarker for early cancer diagnosis or unfavorable prognosis [10,13,17]. Previous studies have reported that KLK6 mRNA has a clinical utility and a prognostic value, as a biomarker in colorectal adenocarcinoma since its expression in CRC correlated significantly with tumor stage and tumor grade and advanced Dukes’ stage, liver metastasis, and poor prognosis [18,19]. Upregulation of KLK6 correlated also with the depth of tumor invasion, presence of distant metastasis, and as an independent marker to predict poor disease-free and survival rate in CRC patients [20]. KLK6 mRNA presence in lymph nodes is a better prediction marker than the traditional marker CEA (carcinoembryonic antigen) [21]. Recently, The Cancer Genome Atlas (TCGA) RNA-Sequencing data analysis of kallikrein gene expression across 15 different cancers highlighted KLK6, KLK7, KLK8, and KLK10 as good candidates for colon adenocarcinoma potential diagnostic biomarkers [22]. Interestingly, analysis of gene expression from TCGA showed that KLK6 may be a suitable prognostic predictor for colon adenocarcinoma [23]. The role of KLK6 in colorectal cancer and the critical functions of KLK6 enzyme in CRC advancement to the late stages have been demonstrated in vitro and in vivo [24]. Recent correlation analysis of RNA-Seq data from the TCGA database in CRC patients with elevated KLK6 transcripts identified members of KLK family—KLK7, KLK8, and KLK10—as KLK6-interacting partners. Other genes, which were highly co-expressed with KLK6, were correlated with signal transduction, extracellular matrix organization, and cell communication pathways, which contribute to cell invasion and metastasis [25]. Despite the evidence that support the critical role of KLK6 in malignant progression of colon cancer the exact involvement of KLK6 in the different steps of metastasis remains unknown. Herein, we demonstrate the expression and secretion of KLK6 in colon cancer cell lines and in CRC tumors resected from patients in contrast to the absence of KLK6 expression in normal colorectal tissues. Furthermore, we demonstrate that KLK6 is an efficient activator of PAR2 signaling leading to ERK1/2 activation. Moreover, KLK6 suppression in the human colon cancer-derived cell line, HCT116 decreased colony formation and increased cell adhesion to extracellular matrix binding proteins. Furthermore, KLK6 suppression in non-adherent conditions (cell culture which mimics the ascites microenvironment) reduced spheroid formation. The KLK6 effects seen in vitro were supported by our small pilot study of patients’ data as we found increased expression of KLK6 in human colon adenocarcinomas, but not in normal epithelia; and high levels of KLK6 protein in the ascites of CRC patients with peritoneal metastasis, but not in benign ascites. Thus, KLK6 seems to promote an aggressive phenotype of colon cancer cells and deserves further evaluation as a differential marker for benign and malignant ascites.

## 2. Materials and Methods

### 2.1. Reagents

Reagents were obtained from the following sources: the activating peptides TFLLR-NH_2_ (AP1, a PAR1 agonist), and SLIGKV-NH_2_ (AP2, a PAR2 agonist) from NeoMPS (Strasbourg, France); Fura-2-AM from, Molecular Probes (Leiden, The Netherlands); purified human recombinant KLK6 from R&D systems (Lille, France). KLK6 was activated according to the manufacturer’s protocol. KLK6 parameters were evaluated against the synthetic substrate Boc-Gln-Ala-Arg-AMC (Bachem, Germany). KLK6 also cleaved efficiently the substrates mimicking PAR1 and PAR2 N-terminal peptides (Data not shown); human α-thrombin (3000 U/mg) from Stago BNL (Lille, France); pancreatic porcine trypsin (16,000 U/mg) from Sigma Aldrich (Saint-Quentin-Fallavier, France); pyromycin, DMEM, RPMI 1640, and HAM’s F12 medium were purchased from Life Techonologies (Cergy-Pontoise, France); human KLK6 polyclonal antibody from Boster (Pleasanton, CA, USA), monoclonal anti-PAR2 (SAM11) (# SC-13504)(Clinisciences, Nanterre, France) and anti-monoclonal PAR1 (WEDE15) (#PN IM2085°) from Beckman Coulter (Marseille, France) and phospho-specific antibodies to ERK1/2 (Thr202/Tyr204) (#9106) from Cell Signaling Technologies (Beverly, MA, USA); anti-ERK1/2 (#SC-94) antibodies from Santa Cruz Biotechnology (Dallas, TX, USA); peroxidase-conjugated anti-mouse (#115-035-068) and anti-rabbit (#111-035-144) antibodies were from *Jackson Immuno-Research* (West Grove, PA, USA). Alexa Fluor^®^ 488 anti-rabbit and anti-mouse IgG from Invitrogen (Carlsbad, CA, USA). ECM Cell Adhesion Array Kit from Millipore, (Saint-Quentin-Fallavier, France).

### 2.2. Cell Culture

The human colon cancer cell lines were obtained from American Type Culture Collection (ATTC) (Rockville, MD). The HT-29-D4 cell line was a gift from Dr. J. Marvaldi (Université d’Aix-Marseille, France). The main characteristics of the cell lines used in this study and the culture conditions have been previously described [24,26]. All cell lines retained their original morphology and growth characteristics. HCT116-control shRNA and HCT116-KLK6 shRNA cells were previously established [24,26].

Normal human colorectal tissue collection has been previously described according to French bioethics law [27,28].

### 2.3. Reverse Transcription Polymerase Chain Reaction (RT-PCR)

RNA was isolated from the cells using Nucleospin RNA kit (Macherey–Nagel, Durem, Germany) according to the manufacturer’s instructions. Four micrograms of total RNA were reverse-transcribed as previously described [14,15]). Residual DNA contaminations from the samples, were removed by DNase treatments during RNA purification. Primer sequences (that anneal to different exons-positions), conditions, and product size are described in Table S1 (Supplementary Table S1). GAPDH cDNA amplification was used as an internal control. The absence of sample DNA contamination was tested by performing reactions without reverse transcriptase. For negative control reactions, we replaced the cDNA by water. After 30 cycles of amplification, PCR products were identified by electrophoresis in 2% agarose gels followed by SYBR^®^Safe staining (Invitrogen, Carlsbad, CA, USA).

### 2.4. Enzyme-Linked Immunosorbent Assay (ELISA) for KLK6

Colon cancer cells were (40,000 cells) were plated in 12 well plates. At confluence, the conditioned medium was collected for antigen determination of KLK6 as described before [14]. Protein values represent the mean concentration of KLK6 expressed by 10^6^ cells. The KLK6 assay has a detection limit of 0.05 ng/mL and a dynamic range up to 5 ng/mL. Precision was less than 15% within the measurement range. Inter-assay variation is <15% and intra-assay variation is <10%. For ascitic fluids, protein values represent the mean concentration of KLK6 per liter.

### 2.5. Intracellular Calcium Measurement

Intracellular calcium concentration was measured using Fura-2/AM as previously described [6,7]. Cells were treated with KLK6, the PAR agonist peptides AP1 (TFFLR-NH_2_) and AP2 (SLIGKV-NH_2_) and changes in intracellular Ca^2+^ were monitored. Fluorescence was measured using a dual-wavelength excitation fluorimeter at 340 and 380 nm for excitation and 510 nm for emission.

### 2.6. Immunofluorescence Staining

HT 29 cells seeded on a coverslip and let grown over night until 70–80% confluency was reached. Cells were fixed in 2% paraformaldehyde, and permeabilized with 0.1% Triton X-100, then cells were blocked with 5% BSA in TBS for 15 min at room temperature prior to application of the primary anti-KLK6 (1:50), anti-PAR1 antibodies (WEDE15) (1:100) or anti-PAR2 antibodies (Sam11) (1:50) at room temperature. All washing steps were performed using PBS. Subsequently, cells were incubated for 45 min with the secondary antibody goat anti-mouse or anti rabbit IgG-coupled to Alexa-Fluor^TM^ 488. Negative controls were obtained by omitting primary antibodies.

We also performed immunofluorescence studies following incubation of HT-29 cells for 20 min at 37 °C with KLK6 (20 nmol/L). The coverslips were mounted in Vectashield medium containing DAPI Dye (Vector, Peterborough, UK) and examined using a fluorescence microscope (Zeiss, Jena, Germany) and by confocal microscopy analysis (LSM 510 Zeiss; (Carl Zeiss QEC GmbH, Germany). Magnification 630X).

### 2.7. Western Blot Analysis

HT29 and HCT116 cells were serum starved and treated for 5 min with active recombinant KLK6 at different concentrations as indicated in the Results section.

Cell lysates were generated using RIPA buffer as described [8]. 25 µg of the RIPA lysates were separated by SDS-PAGE and transferred onto a nitrocellulose membrane and blocked with 5% skim milk in 20 mM Tris, 50 mM NaCl and 0.1% (v/v) Tween 20 and then probed with a monoclonal phospho-ERK1/2 (1:2000) overnight at 4 °C. Membranes were stripped and reprobed with a polyclonal anti-ERK1/2 antibody (1:1000) that recognizes total ERK1/2 regardless of its phosphorylation state as loading controls. HRP-labeled anti-mouse-IgG-antibodies or anti-rabbit-IgG-antibodies were used for detection, respectively. Proteins were revealed by applying the Signal^®^ Chemiluminescent Substrate (Thermo Scientific) on an Image-Quant imaging system.

### 2.8. Colony Formation Assay

KLK6-knock out cells (HCT116-KLK6 shRNA) or vector-control cells (HCT116-control shRNA) described elsewhere [24,26] were detached into single-cell suspensions, plated at 1000 cells/well and allowed to grow in single colonies for 14 days. The colonies were washed in PBS, then stained with 0.5% (W/V) crystal violet–20% methanol, imaged using an *Azure*™ Imaging Systems and the number of colonies was quantified with the ImageJ software V1.8.0 (GE Healthcare, Piscataway, NJ, USA). Columns represent the mean percentage of colonies of three independent experiments, each performed in duplicate.

### 2.9. Cell Adhesion Assay

Cells were seeded at 10^5^ cells/well in triplicates, following instructions by the manufacturer, in 96-well ECM Cell Adhesion plates (Millipore, Darmstadt, Germany) coated with type I collagen (Col-I), type II collagen (Col-II), type IV collagen (Col-IV), fibronectin (FN), laminin (LN), tenascin (TN), or BSA (B) as a negative control cells and incubated for 2 h at 37 °C. Non-adherent cells were washed three times with PBS, the attached cells were then stained using the ECM Cell Adhesion Array Kit and quantified by absorbance readings in a microplate reader at 570 nm. The assay was performed in triplicates. Means of three independent experiments are presented.

### 2.10. Spheroid Formation

Cells were seeded at clonal density of 1000 cells/mL in ultra-low attachment 6-well plates (ThermoFisher^TM^ Scientific, Fontenay-sous-Bois, France) under neural crest cell culture conditions: DMEM GlutaMAX (0.5X; Gibco^®^), F12 NutMix GlutaMAX (0.5X; Gibco^®^), B27 supplement (1X; Gibco^®^, Waltham, MA, USA), antibiotics (100 U/mL penicillin and 1000 μg/mL streptomycin; Gibco^®^, Waltham, MA, USA), murine EGF (20 ng/mL; peproTech, Rocky Hill, NJ, USA), murine bFGF (20 ng/mL; PeproTech, Rocky Hill, NJ, USA), and insulin (5 μg/mL; Sigma Aldrich Chimie, Saint-Quentin-Fallavier, France).

Cells were dispersed in low-adherent plates and medium was changed twice a week. Spheroids were allowed to grow over the course of 12 days. Spheres were counted and measured every 2–3 days under the microscope.

### 2.11. Tissue Immunohistochemistry

Immunohistochemistry (IH) was performed on standard 4-micron formalin-fixed paraffin-embedded tissue samples (n = 37) (Pathology Department of Bichat-Claude Bernard Hospital, Paris). The study was conducted with the approvement of the Human Research Committee of the Bichat-Claude Bernard Hospital and was conducted following the Helsinki Declaration as adopted by the French Bioethical Law [27,28]. Clinicopathological profiles of 37 patients analyzed in this study are presented in Table 1. The patients included 20 males and 17 females with a mean age of 66.2 years (range, 42–91 years) without any treatment before surgery.

KLK6 immunostaining was performed using a Leica Bond Max Automated IHC/ISH Stainer and the Bond Polymer Refine Detection Kit (Leica Microsystems Inc., Nanterre, France) according to manufacturer’s instructions.

Briefly, after heat-based antigen retrieval employing a high-pH (pH, 9) buffer Bond Retrieval Solution at 100 °C, anti-human KLK6 polyclonal antibody was applied at a 1:250 dilution. To detect the primary, a polymeric secondary kit was used. Slides stained with only secondary rabbit IgG antibody served as negative controls. (Detection was performed using DAB (3,3’-diaminobenzidine tetrahydrochloride) Refine. Staining was assessed by two independent pathologists employing a semi-quantitative methodology: first, the percentage of KLK6 immunostained cancer cells was evaluated and second, the staining intensity was scored on a scale between no (0), weak (+1), moderate (+2), and strong (+3) staining. When staining intensity varies, staining average for each case is calculated. Result scores were obtained by multiplying the percentage of positive cells by the average of intensity of the staining.

### 2.12. Peritoneal Fluid

Ascitic fluids (n = 16) were obtained from adult patients with peritoneal metastasis from colorectal cancer (C-PM), gastric cancer (G-PM), or from non-malignant liver diseases taken as control samples (liver cirrhosis (n = 2), hepatic insufficiency (n = 1), or inflammatory complication post-surgery (n = 1), (Department of Surgery, Lariboisière Hospital, Paris, France). Samples were used in accordance with the requirement of the Human Research Committee of Lariboisière Hospital and according to French bioethics law [27,28]. The patients were consented for the samples collection and all individual patient-related reports and data were de-identified before being processed for the samples biobanking. Because all information related to the used patients’ material was de-identified and did not include any protected health information (PHI), it is not considered clinical research according to the Human Subjects Protection Program (HSPP) determination. Ascitic fluid extraction is a part of the routine management of patients, and informed consent, for ascites analysis, was obtained from each patient prior to surgery. The peritoneal fluids obtained were submitted to a short spin at 1000 rpm for 5 min to separate the cells from ascites supernatants. The supernatants were aliquoted and stored for further use at −80 °C.

### 2.13. Statistical Analysis

Results are expressed as mean ± SEM. Analysis were performed using Prism 6 (GraphPad Software Inc., La Jolla, CA, USA), and a one-way ANOVA test or unpaired *t*-test was used for statistical analysis. A *p*-value < 0.05 was considered statistically significant (NS > 0.05, * *p* < 0.05, ** *p* < 0.01, *** *p* < 0.001).

## 3. Results

### 3.1. Expression Patterns of KLKs in Human Colon Cancer Cell Lines

**Figure 1:**
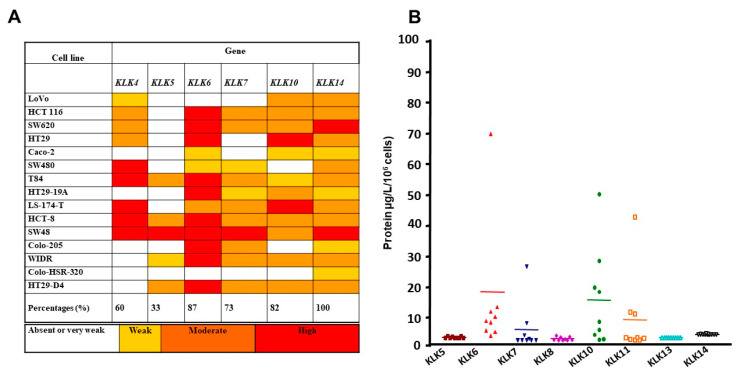
Analysis of the mRNA and protein expression pattern of KLK4, 5, 6, 7, and 14 in different colon cancer cell lines. (**A**) Analysis of KLK mRNA expression levels in the indicated human colon cancer cell lines by semiquantitative RT-PCR. (**B**) Secretion of KLK 4, 5, 6, 7, 8, 10, 13, and 14 in conditioned medium from nine colon cancer cell lines was assessed by ELISA and is presented in a scatter plot. The protein levels are shown as the mean concentration of KLKs (µg/L) secreted by 10^6^ cells per 24 h. Median values are marked with a horizontal line.

We investigated the expression pattern of certain KLKs in colon cancer cells that have been shown individually to be potential prognostic markers for CRC patients [11]. Figure 1 shows that KLK4, KLK5, KLK6, KLK7, KLK10, and KLK14 mRNA were detected in 60%, 33%, 87%, 73%, 82%, and 100% of the analyzed cell lines, respectively (for a summary of the expression pattern of these KLKs see Figure 1A). Thus, colon cancer cell lines co-express many KLK genes. Since KLKs mRNA levels do not always reflect the protein levels [10], we investigated secretion of these proteins into the conditioned media from nine human colon cancer cell lines. The concentrations of KLK5-8, KLK10, KLK11, KLK13, and KLK14 in the cell line conditioned medium were quantified using a non-competitive immunoassay as previously described [14]. As shown in Figure 1B, many KLKs were detected in the supernatants of colon cancer cells. These data suggest that colon cancer cell lines express and secrete distinct amounts of KLKs that may potentially act in an autocrine manner and play a role in colon cancer progression. We focused on KLK6 that was among the KLKs whose expression and secretion by human colon cell lines were high (Figure 1B).

### 3.2. Kallikrein 6 Expression in Human Colon Cancer Cell Lines

**Figure 2:**
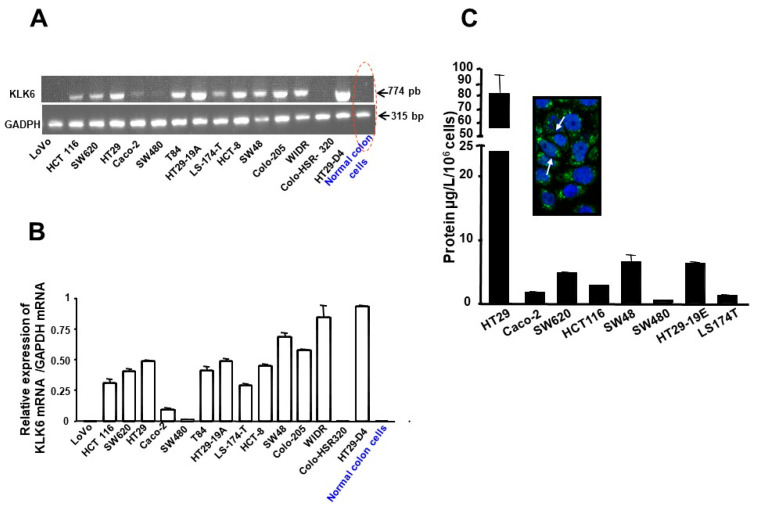
Expression of KLK6 in human colon cancer cell lines. (**A**) Two micrograms of total RNA were reverse transcribed and PCR-amplified with KLK6 or GAPDH primers as described in Material and Methods. A major single PCR-amplified product of the predicted size (774 bp) for KLK6 was visualized after electrophoresis on a 2% agarose gel. GADPH was used as an internal control. Normal isolated epithelial cells do not express KLK 6 mRNA. Note that KLK 6 is present in SW620, a cell line from a lymph node of a primary adenocarcinoma from which SW480 was derived. (**B**) The amount of mRNA expression was quantified by densitometry of bands in comparison to the glyceraldehyde-3-phosphate dehydrogenase (GAPDH). Densitometry of mRNA bands were quantified from three independent PCR experiments presented as mean ± SEM. (**C**) Immunodetection of kallikrein-related peptidases 6 by colon cancer cell lines: Supernatants were collected from colon cancer cells in culture and KLK6 expression was estimated by sandwich-type ELISA (see Material and Methods). Protein values represent the mean concentration of KLK6 (µg/L) secreted by 10^6^ cells, which were cultured for 24 h. Inset: shows confocal microscopic immunocytochemical localization of KLK6 in HT-29 cells (Magnification X630). Arrows show cytoplasmic, perinuclear staining of KLK6.

KLK6 mRNA transcript levels were investigated in 15 human colon cancer cell lines by RT-PCR analysis. As shown in Figure 2, 11 out of 15 analyzed human colon cancer cells expressed KLK6 mRNA (Figure 1A and Figure 2A). A strong signal of KLK6 mRNA was detected at the predicted band size of 764 bp in HT-29, HT-29-D4, Colo-205, HCT8, SW620, SW48, HCT116, T84, and WIDR cells, and the signal was low in LS174T and Caco-2 (Figure 2A). The expression of KLK6 was undetectable in LoVo, SW480, and Colo-HSR320 cells. Interestingly, under the experimental conditions, KLK6 mRNA was not detectable in epithelial cells isolated from normal human colon (Figure 2B).

We did not find any major differences in the KLK6 expression patterns in the primary colon cancer cell lines when compared to the metastatic cell lines (Supplementary Table S2). In addition, no difference in KLK6 expression was found among the cell lines carrying either BRAF or *Kirsten rat sarcoma (K-RAS*) mutations, (which are the frequently mutated oncogenes in colon cancer Supplementary Table S3) [29].

To provide further evidence of KLK6 expression at the protein level, immunofluorescence detection was carried out on HT-29 cells using a polyclonal antibody directed against human KLK6. Confocal laser scanning microscopy showed a strong cytoplasmic staining of KLK6 in HT-29 cells. The immunoreactivity was localized in a perinuclear region of HT-29 cells (Figure 2C-Inset). In contrast, no fluorescence was detected when KLK6 antibody was omitted (not shown). These data are consistent with the RT-PCR detection of KLK6 in HT-29 cells and show clearly that colon cancer cells express high levels of KLK6 protein.

The levels of secreted KLK6 were quantified in supernatants of various cell line by ELISA. As shown in Figure 2C, KLK6 is secreted by many human colon cancer cells tested (Figure 2C). The highest KLK6 levels (95.8 +/− 106 µg/L/10^6^ cells; ~4 nmol/L) were observed in the conditioned media from HT-29 cells followed by HT-29-19E, SW48, SW620, and HCT116; KLK6 immunoreactivity was low in Caco-2, LS174T, and SW480 and undetectable by this method in T84 and LoVo (not shown). Taken together, these data reveal that many colon cancer-derived cell lines express and secrete immunoreactive KLK6 that may potentially act in the extracellular environment in an autocrine manner and play a role in colon cancer progression.

### 3.3. KLK6 Induced Loss of PAR1 and PAR2 from the Cell Surface of Colon Cancer-Derived Cell Line HT29

Recent studies have indicated that many KLKs function as endogenous activators of PARs by promoting the proteolytic cleavage of PAR N-terminal sequences to reveal an activating tethered ligand [16], which activates signal transduction pathways leading to cell proliferation [10,14,30]. Therefore, we investigated whether KLK6 can signal through PAR1 and/or PAR2 in colon cancer cells. The colon cancer-derived cell line HT-29 which expresses both PAR1 and PAR2 was chosen for this analysis [6,7]. By using immunofluorescence microscopy with antibodies directed against the N–terminal domains of either PAR1 or PAR2, we examined the loss of PAR1 and PAR2 immunoreactivity at the cell surface of KLK6-treated HT-29 cells.

**Figure 3:**
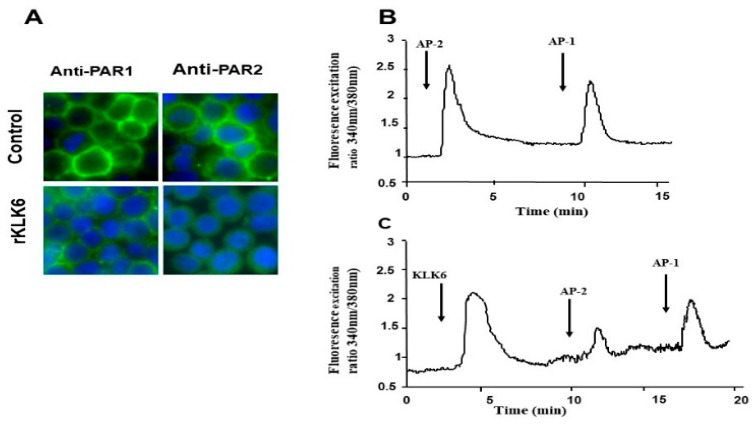
KLK6 induces loss of PAR1 and PAR2 from the surface of HT-29 cells and initiates calcium signaling via PAR2. (**A**) Immunofluorescence detection of PAR1 and PAR2 in HT-29 cells pre-treated with KLK6 (20 nmol/L), or with vehicle (control) for 15 min at 37 °C. Cells were fixed using 2% paraformaldehyde and immunostained with a PAR2 monoclonal antibody or PAR1 monoclonal antibody. Results are representative of two independent experiments (Magnification ×630). (**B**,**C**) KLK6 initiates intracellular Ca^2+^ mobilization. HT-29 cells were loaded for 60 min at 37 °C using Fura 2/AM. (**B**) HT-29 cells were challenged first with AP2 (SLIGKV-NH_2_, 100 µmol/L)) and AP1 (TFFLLR-NH_2_, 100 µmol/L). (**C**) Cells were first challenged with KLK6 (1 µmol/L) followed by a second challenge with activating peptide AP2 (100 µmol/L) and subsequent challenge with AP1 (100 µmol/L). Note the AP2 response showed a reduction in response, whereas cells were still responsive to AP1. Administration of compounds is indicated with the corresponding arrows.

As shown in Figure 3, PAR1 and PAR2 were readily detected at the plasma membrane of unstimulated HT-29 cells (control). Interestingly, stimulation of HT-29 cells with KLK6 (20 nmol/L) for 20 min, resulted in a significant decrease in PAR1 and PAR2 staining at the cell surface and showed diffuse localization of these receptors in the cytosol of HT-29 cells (Figure 3A). This loss of immunoreactivity indicates the receptor cleavage and, possibly, their activation and internalization [14]. Receptor cleavage upstream or downstream of the activation site is also possible, the latter process is often referred to as receptor disarming [16].

### 3.4. Calcium Signaling Triggered by KLK6 Is Mediated by PAR2 but Not by PAR1

The proteolytic cleavage at a specific activation site and exposure of self-activating tethered ligand of PARs induces intracellular calcium mobilization [5]. Therefore, we investigated whether KLK6 can trigger calcium cell signaling in HT-29 cells. As we have previously reported [6,7], receptor selective peptide agonists for PAR1 (TFFLR-NH_2_) and PAR2 (SLIGKV-NH_2_) both induced changes in Ca^2+^ mobilization in HT-29 cells, confirming that that PAR1 and PAR2 are functional. The specificity of the response via PAR1 and PAR2 was demonstrated by cross desensitization studies using specific agonist peptides as ligands [6,7]. As shown in Figure 3B, a significant increase in transient Ca^2+^ mobilization was seen following treatment with 1 µmol/L of KLK6. In concordance with the immunofluorescence data, KLK6-induced calcium mobilization did not abrogate the PAR1 calcium responses induced by AP1 (100 µmol/L). KLK6 abrogated the PAR2-Ca^2+^ response when cells were challenged with AP2 the known agonist of PAR2 whereas the subsequent challenge of HT29 cells with AP1 (TFFLR-NH2) (100 mol/L) still induced Ca^2+^ elevation (Figure 3C). As a control, when inactivated (heat-treated at 60 °C for 20 min) KLK6 was added to HT-29 cells, no transient Ca^2+^ mobilization was seen. These results suggest that KLK6 preferentially activates PAR-2 in HT29 cells.

### 3.5. KLK6 Protein Induces ERK1/2 Phosphorylation in Human Colon Cancer Cells

**Figure 4:**
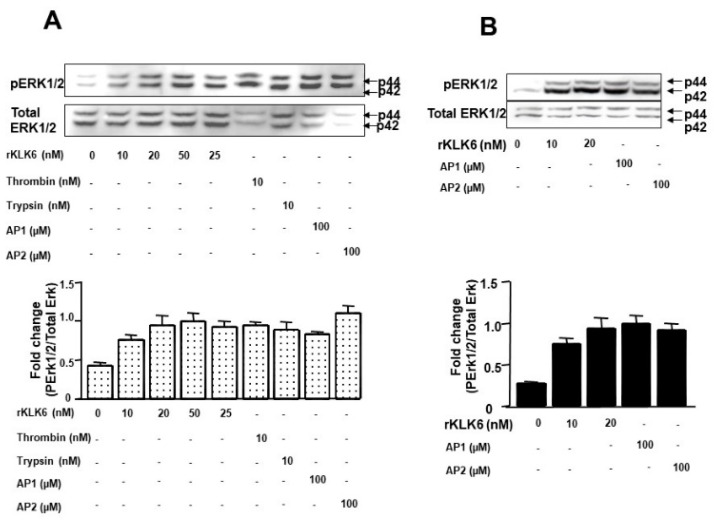
Dose-dependent activation of p42/p44 MAP-Kinase phosphorylation by KLK6. (**A**) Quiescent HT-29 cells were stimulated with the indicated concentrations of KLK6, thrombin (0.01 µmol/L), trypsin (0.01 µmol/L), with 100 µM of TFLLR-NH_2_ (AP1, a PAR1 agonist), 100 µM SLIGKV-NH_2_ (AP2, a PAR2 agonist) or with vehicle (control) for 5 min. (**B**) Quiescent HCT116 cells were stimulated with the indicated concentrations of KLK6, with 100 µM of TFLLR-NH_2_ (AP1, a PAR1 agonist), with 100 µM SLIGKV-NH_2_ (AP2, a PAR2 agonist) or with vehicle (control) for 5 min. To confirm equal protein loading, the membranes were stripped and incubated with p42/p44 MAP-Kinase antibody. p42/p44 MAPKinase protein seems to be reduced in PARs agonist-stimulated samples, possibly because the high signal with the anti-phopsho-ERK1/2 (Thr202/Tyr204) antibody prevents subsequent anti-ERK1/2 antibody binding to the ERK1/2 epitope as observed previously [9]. Densitometric analysis of the phospo-p42/p44 MAP-Kinase divided by total amount of p42/p44 MAP-Kinase is represented in lower panels. Results are representative of two separate experiments.

Since we have previously shown that PAR activation plays a pivotal role in extracellular-regulated kinase (ERK1/2)-induced activity in colon cancer [7,8,9], we next investigated the effect of KLK6 on ERK1/2 phosphorylation in HT-29 cells. Treatment of HT-29 cells with KLK6 for 5 min induced ERK1/2 phosphorylation in the range of concentrations between 10 nmol/L and 50 nmol/L (Figure 4A). Significant ERK1/2 phosphorylation was observed with KLK6 concentrations as low as 10 nmol/L, reaching a maximum within the range of concentrations between 20 nmol/L and 50 nmol/L. At 50 nmol/L KLK6 induced a signal comparable with 10 nmol/L of thrombin or trypsin and with 100 µmol/L AP1 or AP2 (Figure 4B). Trypsin and thrombin were used here at concentration of 20 nM as positive controls for ERK1/2 phosphorylation.

We also tested the effect of exogenous KLK6 on the HCT116 cell line (used in our experiments described below). Again, KLK6 induced ERK1/2 phosphorylation in the same range of concentrations (Figure 4B). These experiments suggest that KLK6 activates the MAP-kinase pathway in colon cancer cells at the concentrations equivalent to those of PARs agonists [6,7]. These findings indicate that KLK6 can activate the MAP-kinase pathway in colon cancer cells and plays an important role in colon tumorigenesis.

### 3.6. KLK6 Silencing in HCT116 Cells Reduced Colony Formation

**Figure 5:**
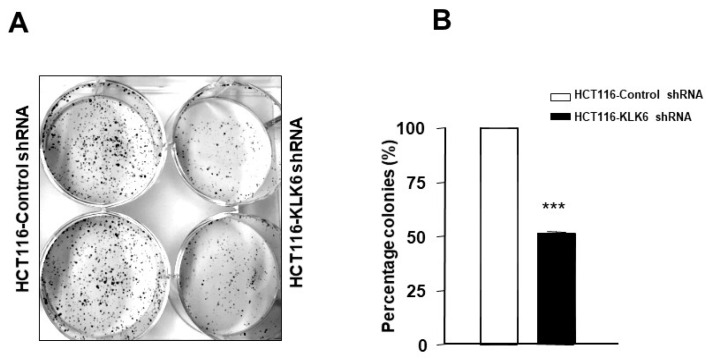
The effect of KLK6 knockdown on colony formation. (**A**) HCT116-control shRNA and HCT116-KLK6 shRNA were plated as described in Materials and Methods and incubated for 12 days. Silencing KLK6 induced a strong decrease in colon cancer colony formation. Representative images were captured and quantified using an Azure™ Imaging Systems. (**B**) Analysis of colony formation rates is shown as percentage of colonies from three independent experiments, each performed in duplicate. Data are shown as mean ± SEM. *** *p* < 0.001.

Colony formation assay was performed to investigate long-term effects of KLK6 on a single cell ability to reproduce and form a colony. We employed HCT116 isogenic colon cancer cell model with the negative control cells (HCT116-control shRNA) and cells with knockdown expression of KLK6 (HCT116-KLK6 shRNA cells) and followed cells in culture for 2 weeks. The colony forming ability was significantly suppressed in HCT116-KLK6 shRNA-Clone-3, the most effective KLK6 knockdown clone [26] (*p* < 0.001, when compared to HCT116-control shRNA) (Figure 5). This finding was confirmed using other HCT116-KLK6 shRNA clones. These data indicate that KLK6 supports the colony formation capability of colon cancer cells through its regulation of cell–cell communication and cell adhesion genes, as previously suggested [26].

### 3.7. KLK6 Silencing in HCT116 Cells Alters Cell Adhesion

**Figure 6:**
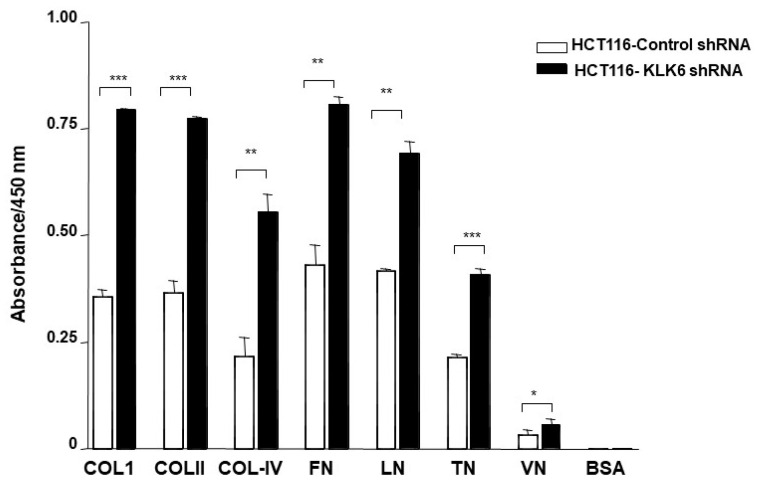
Effect of KLK6 knock down on cell adhesion. The adhesion assay was performed by seeding HCT116-KLK6-KO and HCT116-control cells (10^5^ cells each) in 96-well plates coated with type I collagen (Col-I), type II collagen (Col-II), type IV collagen (Col-IV), fibronectin (FN), laminin (LN), tenascin (TN), or BSA as a negative control. Cells were incubated for 2 h. Non-adherent cells were washed three times with PBS, the attached cells were then stained using the ECM Cell Adhesion Array Kit and quantified by absorbance readings in a microplate reader at 570 nm. Means of three independent experiments are presented. Data are shown as mean ± SEM. * *p* < 0.05; ** *p* < 0.01; *** *p* < 0.001.

Interactions between the cancer cells and extracellular substrates are essential for cell growth, migration, and survival during the process of tumor development and spreading. To examine whether KLK6 could, in fact, affect adhesion to the extracelular matrix (ECM), adhesion assays were performed using selected components of the basement membrane and ECM—i.e., type I, II, IV collagens (Col-I, Col-II, Col-IV), fibronectin (FN), laminin (LN), and vitronectin (VN). As shown in Figure 6, regardless of the absolute numbers of cells attached to the individual ECM component, HCT116–KLK6 shRNA cells showed a significant increased cell adhesion to all ECM proteins (Figure 6). These results support a role for KLK6 in altering adhesion abilities of colon cancer cells.

### 3.8. KLK6 Effect on Spheroid Forming Ability of Colon Cancer Cells

**Figure 7:**
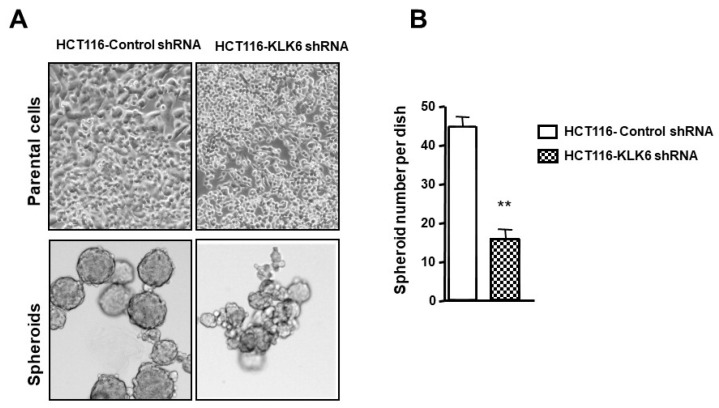
Effect of KLK6 silencing on colon cancer spheroid formation. Spheres were generated by growing cells at a clonal density of 1000 cells/mL in neural crest cells culture conditions as described in Material and Methods. Cells were dispensed in low-adherent plates and medium was changed twice a week. Spheres were allowed to grow over the course of 12 days. Spheres were counted and measured every 2–3 days under the microscope. (**A**) Upper panel: KLK6 silencing results in altered cell morphology. Lower panel: representative images of spheroids formed by HCT116-KLK6 shRNA and HCT116-control shRNA cells randomly taken at day 6 after subculture. (**B**) Spheroids number formed by HCT116-KLK6 shRNA and HCT116-control shRNA cells. Data are shown as mean ± SEM of three independent experiments each performed in duplicate. ** *p* < 0.01.

In adherent culture, KLK6 suppression induced a loose non-compact phenotype of HCT116 cells (Figure 7A, upper panel) compared to HCT116-control cells. Therefore, we hypothesized that KLK6 may enhance aggregation of colon cancer cells in non-adherent conditions. Although both HCT116-control shRNA cells and HCT116-KLK6 shRNA cells were able to form spheroids in non-adherent conditions, HCT116-control cells which express high levels of KLK6 showed about a three-fold increase in spheroid numbers, reaching approximately 50 spheroids within 12 days *(*Figure 7B), compared to HCT116-KLK6 shRNA cells. In addition, knocking-down KLK6 in HCT116 cells resulted in a dramatic loss of spheroid structure formation and organization compared to spheroids formed from control cells (Figure 7A, lower panel). These data suggest that KLK6 promotes formation of colon cancer multicellular spheroids that may enable tumor cell survival during dissemination.

### 3.9. KLK6 Is Expressed in Colon Cancer Tumors In Vivo

**Figure 8:**
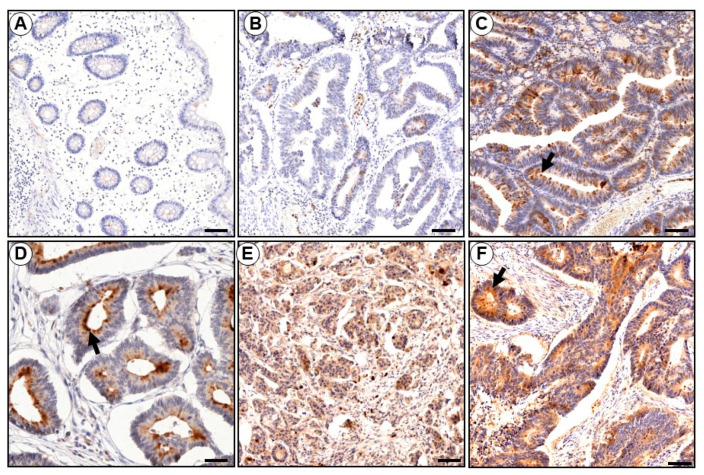
Representative immunostaining of the KLK6 in paraffin sections of colonic tissues from patients with adenocarcinoma. (**A**) KLK6 immunoreactivity is absent in ‘normal-appearing’ colonic mucosa very distant (more than 10 cm) from a colon adenocarcinoma (**B**) Immunostaining for KLK6 in the dysplastic colonic mucosa from the same patient as in (**A**) is seen. (**C**–**F**) Moderate to strong positive immunoreactivity in the adenocarcinomas of four different patients. KLK6 immunoreactivity appeared to be distinguishable into a predominantly apical/cytoplasmic pattern (arrows). Bar = 100 µm (**A**–**F**), 10 µm (**D**).

To evaluate expression of KLK6 in colonic tumors in vivo, we next performed IHC analysis of KLK6 expression in colon cancer samples from patients with adenocarcinomas and normal colonic epithelium. As shown in Figure 8, minimal if any staining for KLK6 was observed in epithelial cells of the normal appearing human colonic mucosa resected at more than 10 cm away from the neoplastic colon cancer lesions (Figure 8A). Normal mucosa from control colonic tissues did not stain at all with the KLK6 antibody (data not shown). The KLK6 staining clearly appeared in the low-grade dysplastic mucosa. No immunoreactivity was detected in the low-grade dysplastic (Figure 8B) colonic mucosa contiguous to the apparently normal mucosa (Figure 8A). Strong KLK6 staining was clearly seen in the mild dysplastic colonic mucosa (Figure 8C,D) and increased in intensity was observed as the dysplasia progressed to cancer (Figure 8E,F). KLK6 staining was localized in the cytoplasmic compartment and appeared strong in the apical part (Figure 8C,D). No KLK6 staining was evident in the stroma in these experimental conditions. The intensity of labeling varied among the different patients’ adenocarcinomas analyzed (Figure 8). No staining was seen in the same tissue sections when the primary antibody was omitted (not shown). Thirty-one out of 37 tumors tested (84%) were positive for KLK6. Table 1 shows the immunoreactivity scores of KLK6 for each tumor in relation with age, gender, tumor stage, histological type, and differentiation grade. It is worth noting that the RT-PCR data agreed with the immunohistochemical data showing a lack of expression of KLK6 in normal tissue vs. its upregulated expression in the colonic adenocarcinoma tissues (Figure 8). These observations confirm that human colonic adenocarcinomas express higher levels of KLK6 than normal colonic mucosa, thus indicating its potential value as a marker for colonic carcinogenesis.

**Table 1:** Estimation of KLK6 expression in colon carcinoma epithelial cells.

Cases	Age	Gender	Tumor Site	Histological Type	Tumors Stage	Differentiation Grade	Percent of Stained Cells	Staining Intensity	Scores
1	77	F	Cecum	Adenocarcinoma	T_3_N_0_ M_X_	3	50%	2	100
2	69	F	Left colon	Adenocarcinoma	T_4_N_0_M_X_	2	0%	0	0
3	70	F	Sigmoid	Adenocarcinoma	T_3_N_0_M_X_	3	80%	2	160
4	63	M	Sigmoid	Adenocarcinoma	T_3_N_0_M_X_	3	0%	0	0
5	60	M	Right colon	Adenocarcinoma	T_3_N_0_M_X_	1	0%	0	0
6	66	M	Sigmoid	Adenocarcinoma	T_3_N_0_M_X_	3	0%	0	0
7	52	F	Sigmoid	Adenocarcinoma	T_3_N_1_M_X_	3	70%	2	140
8	50	F	Right colon	Adenocarcinoma	T_3_N_0_M_X_	3	90%	3	270
9	49	M	Right colon	Adenocarcinoma	T_3_N_1_M_X_	3	100%	1	100
10	54	F	Left colon	Adenocarcinoma	T_3_N_0_M_X_	2	90%	3	270
11	86	F	Sigmoid	Adenocarcinoma	T_4_N_0_M_X_	2	100%	2	200
12	60	M	Rectum	Adenocarcinoma	T_3_N_0_M_X_	3	0%	0	0
13	57	M	Cecum	Adenocarcinoma	T_3_N_0_M_X_	3	100%	2	200
14	78	M	Right colon	Adenocarcinoma	T_3_N1M_X_	3	90%	2	180
15	75	M	Sigmoid	Tubulo-villous adenoma	T_1_N_0_M_X_	3	10%	1	10
16	91	F	Cecum	Mucinous adenocarcinoma	T_3_N_0_M_0_	2	60%	1	60
17	77	M	Sigmoid	Mucinous adenocarcinoma	T_3_N_2_M_x_	2	10%	1	10
18	84	F	Cecum	Adenocarcinoma	T_3_N_0_M_x_	3	40%	1	40
19	85	F	Right colon	Adenocarcinoma	T_3_N_2_M_x_	2	40%	1	40
20	90	F	Cecum	Adenocarcinoma	T_2_N_0_M_x_	3	60%	2	120
21	76	M	Right colon	Adenocarcinoma	T_3_N_1_M_1_	2	10%	3	30
22	81	M	Right colon	Tubulo-villous adenoma	TisN_0_M_X_	3	40%	2	80
23	74	F	Right colon	Adenocarcinoma	T_3_N_1_M_X_	1	90%	3	270
24	67	M	Right colon	Adenocarcinoma	T_3_N_2_M_x_	2	0%	0	0
25	79	M	Right colon	Adenocarcinoma	T_4_N_1_M_X_	2	30%	2	60
26	42	F	Sigmoid	Adenocarcinoma	T_4_N_2_M_1_	3	10%	2	20
27	71	M	Right colon	Adenocarcinoma	T_4_N_1_M_x_	2	50%	1	50
28	89	M	Sigmoid	Adenocarcinoma	T_1_N_0_M_x_	3	95%	3	285
29	72	F	Cecum	Adenocarcinoma	T_2_N_0_M_x_	2	100%	4	400
30	72	F	Left colon	Adenocarcinoma	T_2_N_0_M_x_	2	100%	3	300
31	65	M	Right colon	Tubulo-villous adenoma	T_1_N_0_M_x_	3	20%	2	40
32	69	F	Left colon	Adenocarcinoma	T_3_N_1_M_x_	2	10%	1	10
33	45	F	Left colon	Adenocarcinoma	T_3_N_1_M_x_	3	100%	3	300
34	47	F	Cecum	Adenocarcinoma	T_3_N_1_M_x_	2	80%	3	240
35	60	F	Left colon	Adenocarcinoma	T_3_N_0_M_x_	3	100%	4	400
36	70	M	Right colon	Adenocarcinoma	T_3_N_1_M_x_	2	10%	2	20
37	64	F	Sigmoid	Adenocarcinoma	T_3_N_0_M_x_	2	5%	1	5

Staining intensity was often variable from place to place and scored as: 0, negative; 1, weak; 2, moderate; 3, strong; 4, intense. Differentiation grade is scored as: 1, well; 2, moderately; 3, poorly differentiated. Results of scores were obtained by multiplying the percentage of positive cells by the intensity; Maximum = 400. Tis: carcinoma in situ; T1, T2, T3, and T4 size and or extend of primary tumor. N0, no regional lymph node involvement; N1, N2, N3, and N4 involvement of regional lymph nodes and/or extent of spread. Mx: distant metastasis cannot be evaluated; Mo: no distant metastasis; M1: distant metastasis is present.

### 3.10. KLK6 Identification in Malignant Ascitic Fluids from Peritoneal Metastasis of Colon Cancers

**Figure 9:**
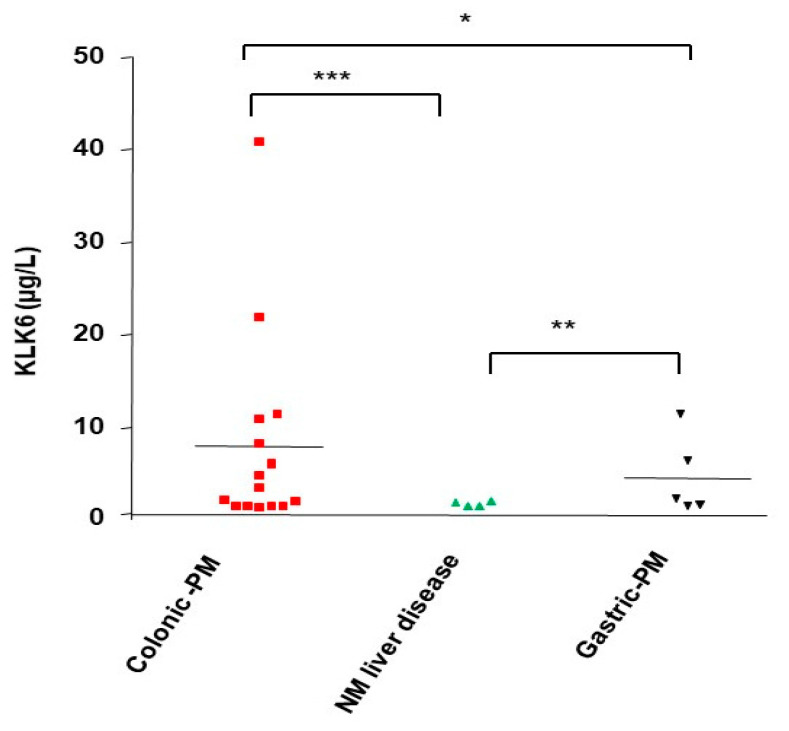
Immunodetection of KLK 6 in ascitic peritoneal metastasis from human colorectal cancer. Ascitic fluids were collected from peritoneal metastasis from human colorectal cancer (Colon-PM), (Gastric-PM) or from non-malignant liver diseases (NM Liver disease) and KLK6 expression was estimated by a sandwich-type ELISA (see Material and Methods). Protein values represent the mean concentration of KLK6 (µg/L). Data are shown as mean ± SEM. * *p* < 0.05; ** *p* < 0.01; *** *p* < 0.001.

Since KLK6 upregulation is associated with the more aggressive tumor phenotype (this study) and poor prognosis [20,25], we investigated if KLK6 could be detected in ascitic fluids from peritoneal metastasis of colon cancers. As shown in Figure 9, high levels of KLK6 (reaching ~40 µg/L) were present in ascitic fluids of several patients with peritoneal metastasis from colon cancers. KLK6 is also expressed in some ascitic fluids from peritoneal metastasis of gastric cancer, but at much lower levels. In contrast, we could not detect any KLK6 in ascitic fluids from non-malignant liver diseases such as cirrosis, liver hepatic insufficiency, or post-surgery inflammation (Figure 9). This has not been explored before and suggests that KLK6 may be involved in the formation of a peritoneal metastatic niche directly or indirectly and may have an important value for discrimination between malignant and non-malignant ascite accumulations.

## 4. Discussion

Human kallikrein-related peptidases s (KLKs) have emerged as regulators of cancer growth, invasion, and metastasis in many cancers including colon cancer [10,11,12,14,24,30]. However, only a limited number of studies have aimed to analyze the precise involvement of KLKs in the metastatic process [25,31,32,33]. In this study, we show that from all the overexpressed KLKs in colon cancer-derived cell lines, KLK6 was found to be among the major highly expressed kallikrein-related peptidases in vitro and capable to control cell proliferation through PAR2 signaling. The influence of KLK6 on cancer cell behavior was demonstrated through the silencing of KLK6 which resulted in a decrease in colony formation, an increase in cell adhesion to ECM, and inhibition of spheroid formation. Patient data support this phenotype as IHC analysis showed ectopic expression of KLK6 in human colon adenocarcinomas but not in normal epithelia. The high levels of KLK6 protein were also detected in the ascites of CRC patients with peritoneal metastasis, but not in benign ascites. Thus, KLK6 seems to promote an aggressive phenotype in colon cancer and may be involved in peritoneal metastasis. These data are in agreement with our recent analysis of KLK6 in the CRC cases from TCGA that shows a direct association between high *KLK6* mRNA levels in colonic tumors and patients’ survival outcomes [25]. Thus, KLK6 is a colon cancer-produced protease that plays a role in the colon cancer aggressive phenotype and in the metastatic process.

In human colon cancer-derived cell lines, KLK6 levels in the supernatant of colon cancer cells were high (up to 70 µg/L), which is in agreement with the levels found in the sera from patients with colon cancer (10–40 μg/L) [34]. KLK6 expression has been examined either individually or using gene/protein panels to evaluate its diagnostic or prognostic value in the different types of cancers as reviewed in [10,12,17]. In human colon cancers, KLK6 mRNA has been extensively studied as a clinical biomarker [17,19,20,35,36]. We show by immunohistochemistry that KLK6 is expressed (84%) in resected colonic tumors from the CRC patients and the mucosa from control normal colonic tissues did not stain at all with the KLK6 antibody. This is in line with the absence of KLK6 mRNA analyzed by RT-PCR in the normal human colon samples. Our findings are in agreement with another report that found that KLK6 mRNA expression is an independent, unfavorable molecular prognostic biomarker in colorectal adenocarcinoma, with an additional prognostic value in patients without regional or distant metastases [20]. Interestingly, we observed a significant clear trend between the intensity of KLK6 and the severity of the lesion. Although we cannot provide significant statistical association, given the limited number of patients, these observations are in line with previous reports showing that high expression of KLK6 mRNA correlated with serosal invasion, liver metastasis, advanced Duke’s stage, poor prognosis, and colorectal cancer patients’ survival [17,19,20,35,36]. Our findings are also in agreement with the in silico analysis showing that KLK6 is absent in normal colonic tissues and aberrantly expressed in colonic tumors [18]. The variability levels of KLK6 mRNA in cancerous than in noncancerous colorectal tissues and the different patterns of KLK6 expression have been reported in some of the tested cell lines [19,36,37] may be explained by the heterogeneity of KLK6 transcripts which continue to be reported [17,38]. Variability could relate also to technical sensitivities (e.g., in silico, qRT-PCR, and ELISA and immunohistochemistry) or to selected tissues (i.e., colon mucosa from healthy donors or paired adjacent mucosa). Indeed, some KLKs are highly expressed at sites of inflammation [39] and might, therefore, also be elevated both in the immune cells and in the glandular epithelial cells in non-cancer-bearing tissues obtained from individuals with non-diagnostic bowel diseases. In our analysis, we did not detect KLK6 in inflammatory cells and KLK6 staining was restricted to the cancerous mucosa. Although aberrant expression of KLK6 was reported in several human cancers [13,17], the mechanism whereby the KLK6 gene is switched on is still poorly understood and is likely to be influenced by multiple mechanisms. As previously reported KLK6 expression in colon cancer can be induced by the major colon cancer driver gene, the oncogenic (*K-RAS*) [40]. Although, K-RAS mutations have been identified as frequently (40%) mutated oncogene in colon cancer [29], in our study we did not find any association between KLK6 and the mutation status in human colon cancer cell-derived cell lines. It is possible that alternative mechanisms could cooperate to influence KLK6 expression. Indeed, in our recent analysis of KLK6 in TCGA and Gene Expression Omnibus databases we observed that high KLK6 expression in colonic tumors correlated to mutations in other genes such as Titin (*TTN), APC*, *K-RAS*, and *MUC16* (CA-125) [25], which are found to be overexpressed or frequently mutated in colon cancer [29]. Another analysis based on the TCGA found that KLK6 overexpression correlated with c-*MYC* expression and both are associated with overall survival of patients with colon cancers [41]. In a recent study, 65-gene signature was screened, which showed a prognostic prediction effect in colon adenocarcinoma. Only KLK6, COL11A1, and WNT2 has been found to be suitable prognostic predictors for colon adenocarcinoma. Ohlsson et al., using a limited number of lymph nodes from CRC patients and combined analysis of CEA and KLK6 mRNAs in lymph nodes, showed that KLK6 was a more sensitive indicator of poor prognosis than carcinoembryonic antigen (CEA) [41]. A combined analysis in a larger cohort of CRCs of KLK6, in the future, with other markers could be a powerful prognostic tool in CRC. In addition, many studies have shown that aberrant expression of KLK6 in human malignancies, including colon cancer, is often regulated by microRNAs interactions [17,26,42]. Moreover, DNA methylation in the proximal promoter has been shown to repress KLK6 expression in breast cancer [43]. KLK6 upregulation in colon cancer may be rather influenced by other factors from the tumor microenvironment. In line with this assumption, TNF-α and IL-17A induced the expression of KLK6, KLK10, KLK11, and KLK13 in NHEKs cells [44]. Further studies are thus warranted to evaluate the role of other pathways in KLK6 induction in colorectal cancers.

Nevertheless, it remains to be determined how the endogenously released KLK6 might contribute to colon tumorigenesis. KLK6 is synthesized and secreted as a zymogen that requires cleavage of a short pro-domain by a trypsin-like protease to become an active enzyme [17]. Many in vitro studies have shown that KLK6 may undergo autoactivation or become activated by other proteases including other KLKs, such as KLK14 and KLK5 [17], for which overexpression in human colon tumors has been demonstrated in our previous report [15] and in ongoing work (unpublished data). It is also conceivable that tumor microenvironmental conditions could play a role in the activation of pro-KLK6, as tumors are known to acidify their microenvironment, which results in the activation of secreted enzymes [45].

As we demonstrated using the in vitro cell survival assay, KLK6 suppression in HCT116 cells reduced the ability of HCT116 cells to form colonies. In line with our data, KLK6 suppression using KLK6-siRNAs led to a reduction in cell proliferation possibly through an antiapoptotic effect of KLK6 [19].

In our immunofluorescence analysis, the signal disappearance of PAR1 and PAR2 in KLK6-treated HT-29 cells reflects either receptor internalization or cleavage downstream of the activation site. The latter process is often referred to as ‘receptor disarming’, as PAR-mediated signaling via calcium mobilization requires proteolytic cleavage at a specific activation site [5]. Further analysis of calcium mobilization in HT-29 cells, that express both PAR1 and PAR2 [8,9], have yielded evidence that KLK6 induces signaling mostly via PAR2. Indeed, the KLK6-induced Ca^2+^ flux significantly reduced the effect of SLIGKV-NH_2_ (AP2, a PAR2 agonist) while the response to TFFLR-NH_2_ (AP1, a PAR1 agonist) remained almost unchanged. Our results are in agreement with two others reports showing that KLK14, KLK5, and KLK6 are strong activators of PAR2 in cells recombinantly expressing these receptors reviewed in [16]. In prostate cancer, lung cancer and oral squamous cell line models KLK6 also signals through PAR2 and not PAR1 as reviewed in [10,12,13]. Several experimental observations suggested that PAR2 function as a positive regulator of tumor growth and/or progression. Indeed, PAR-2 has been reported to be overexpressed in colon cancer cells and its activation lead to cell proliferation and ERK1/2 phosphorylation [6]. PAR2 also modulates transactivation of other cell surface receptors (i.e., EGFR) that are frequent drivers of colorectal tumor progression [8,9]. Thus, KLK6 becomes a potential endogenous PAR2 activator in colon cancer owing to its aberrant expression. To our knowledge, this is the first evidence for a direct link of KLK6 to PAR2 receptor signaling in colorectal tumors.

In adhesion assay, HCT116-control cells showed a reduced adhesion to the individual ECM components compared to HCT116-shKLK6 cells where KLK6 was knocked down. This is in line with in vitro studies that have shown that purified human recombinant KLK6 can efficiently degrade the high-molecular-weight extracellular matrix proteins [17]. In line with our data, the stable combined KLK4–7 overexpression in ovarian cancer cell line reduced cell growth through a reduction in attachment to ECM and integrin downregulation [31]. Interestingly, in our recent analysis of the molecular pathways associated with KLK6 overexpression in colorectal cancer we observed altered expression in laminin receptor and integrin 4B [25]. Whether KLK6 alter adhesion of colon cancer cells via its interaction with integrins or other adhesion molecules will need to be investigated in detail.

Tumor cells have developed a variety of strategies including to overcome chemo-resistance and to grow, invade, and to disseminate into distant tissues. The multicellular tumor spheroid model is an intermediate model between the in vivo tumors and monolayer cultures. In this study, we showed for the first time that KLK6 has a role in colon cancer spheroids formation. Interestingly, trypsin—a potent PAR2 activator [8], with similar substrate cleavage specificity to KLK6—has been reported to induce aggregation of human colon cancer derived cell line, Colo 205 [46]. Our findings suggest that KLK6, by enhancing the multicellular aggregation may facilitate the formation of cancer cell emboli in vivo (in blood vessels) or tumor cell clusters in malignant peritoneal ascites.

Increased cell–cell adhesiveness of metastatic cells allows them to form multicellular homotypic aggregates, which have an increased chance of survival once detached from the primary tumor [2]. Analysis of spheroids in HCT116 cell model showed that knockdown of KLK6 has led to a significant decrease in formed spheroid number compared to the HCT116 control cells that generated uniform compact structure spheroids. Spheroid formation is directed by homotypic cell–cell interaction which is primarily mediated via the adherent junctions proteins such as E-cadherin and many cell surface adhesion proteins [47]. Although the mechanism of KLK6-induced spheroid formation is not yet known, it is unlikely to be mediated by E-cadherin since reported molecular profiling of HCT116 spheroids demonstrated that multicellular aggregation of HCT116 cells in non-adherent conditions is not mediated by E-cadherin, but depends on other adherent molecules [47]. Nevertheless, E-cadherin has been reported to be strongly expressed in primary colorectal cancer tissues, peritoneal metastatic tissues, and cell blocks of malignant ascites of CRC patients with peritoneal carcinomatosis [48]. Therefore, E-cadherin may not be essential for multicellular aggregation in 3D cultures in vitro as it does in tumors in vivo. It has been reported that the concomitant expression of KLK4–7 in 3D ovarian cancer cells drives spheroid formation involving integrins [10,49]. Further studies with ECM/BM preparation in 3D analysis, that mimics in vivo are needed to understand the role of KLK6 in multicellular aggregation and analyze the molecular mechanism associated with spheroid formation.

The presence of peritoneal metastasis in patients with colorectal cancer is associated with an extremely poor prognosis [2]. Our findings highlighted the role of KLK6 in supporting an aggressive phenotype in colon cancer in vitro. Therefore, we analyzed the presence of KLK6 in ascitic fluids from CRC patients with peritoneal metastasis from colon cancers. We found that KLK6 is highly expressed in the malignant ascites of patients with peritoneal metastasis of colon cancer in contrast to it being undetectable in non-malignant ascitic fluids from patients with benign liver diseases. KLK6 was also found in ascitic fluids from patients with peritoneal metastasis from gastric cancers. Given the small number of patients, further analysis will be needed to verify our findings.

Peritoneal metastasis is more frequent and abundant in ovarian cancer where the expression of certain members (KLK6 and KLK10) of the KLK family in ascitic fluids from ovarian cancers has been reported [50]. Notably, the ascitic fluids from colon cancers patients with peritoneal metastasis are less frequent and their volume is very little.

The amount of KLK6 found in the ascitic fluids from patients with peritoneal metastasis of colon cancers (0.2 to 40 ng/mL) were in the same range as the KLK6 amount found in ovarian ascites (42 ng/mL) [51]. Whether KLK6 protein in the ascites of colon metastasis is active or not warrants further investigations that need specific tools [50]. Indeed, Oikonomopoulou et al. found that only a very small proportion of immunoreactive KLK6 in ascites fluids of ovarian cancer is active due to the presence of multiple serine protease inhibitors [50]. Still, localized active KLK6 expression in the ascites of colon cancer patients may play a role in spheroid formation to maintain growth and chemoresistance. It is worth noting that many KLKs can regulate the bioavailability and release of soluble growth factors [12] that can bind to and activate corresponding receptors, which, in turn, modulate cell survival and mitogenesis. These data provide evidence that KLK6 may be involved in colorectal cancer metastasis in the peritoneum. Ascitic fluid analysis is essential for the diagnosis of malignant ascites, and one would speculate that KLK6 expression may have an important value for distinguishing between ascites from malignant colon cancer and ascites, which may develop from benign diseases. Thus, KLK6 may constitute a valuable diagnostic biomarker.

## 5. Conclusions

To conclude, we have demonstrated for the first time the consequences of aberrant KLK6 expression in colon cancer derived cell lines and its link to PAR2 receptor signaling, leading to significant ERK1/2 phosphorylation and cell proliferation, presumably by activating PAR2. KLK6 suppression decreased colony formation, but increased cell adhesion to extracellular matrix binding proteins, whereas KLK6 suppression reduced spheroid formation and compaction. This malignant phenotype is supported by patients’ data as we found ectopic expression of KLK6 in a 37 human CRC samples and its absence in normal epithelia. We also detected the high levels of KLK6 protein in ascites of CRC patients with peritoneal metastasis. Interestingly, ascites from non-malignant disease did not contain detectable KLK6 nor express KLK6. Overall, the present study highlights the role of KLK6 in promoting the aggressive CRC phenotype and suggests the utility of KLK6 for differentiating between benign and malignant ascites. Hence, KLK6 may be further considered as an attractive therapeutic target for the management of aggressive colon cancers.

## Supplementary Materials

The following supporting information can be downloaded at: https://www.mdpi.com/article/10.3390/biom12071003/s1, Table S1: Primer sets used for RT-PCR, the expected amplicons (bp) and annealing temperature. Table S2: Colorectal cancer cell lines origin. Table S3: Subtypes and mutation status of colorectal cancer cell lines. References [52,53,54] are cited in the Supplementary Materials.
