# Supplementary material for: Kallikrein-Related Peptidase 6 (KLK6) as a Contributor toward an Aggressive Cancer Cell Phenotype: A Potential Role in Colon Cancer Peritoneal Metastasis
Source: Biomolecules. 2022 Jul 19;12(7):1003. doi: 10.3390/biom12071003 (PMC9312869; doi:10.3390/biom12071003)
Supplement: Supplementary file 1 [file biomolecules-12-01003-s001.zip › biomolecules-1746502-supplementary.pdf]

Table S1 : Primer sets used for RT-PCR , primer positions, the expected amplicons (bp) and annealing temperature

mRNAs	Primers	Primer position	Product length (bp)	Annealing temperature (°C)
KLK 4	Forward : 5′- GGATCGCTCGTCTCTGGTAG-3′	Exon 1/2	561	60
	Reverse: 5′-AGAGTCACCGTTGCAGGAG-3′	Exon 6		
KLK 5	Forward : 5′-CAAGACCCCCCTGGATGTGG-3′	Exon 2	345	65
	Reverse : 5′-CCGAGACGGACTCTGAAAACTTTCTTCC-3′	Exon 4/5		
KLK 6	Forward : 5′GAAGCTGATGGTGGTGCTGAGTCTG	Exon 5	454	61
	Reverse : 5′AG ACAGCAGATGGTGATTTCCCTGAC	Exon 7		
KLK 7	Forward: 5′-GCCCAGGGTGACAAGATTATT-3′	Exon 3	569	62
	Reverse : 5′-GTACCTCTGCACACCAACGG-3′	Exon 6		
KLK 10	Forward : 5′- GCGGAAACAAGCCACTGTGGG -3′	Exon 3/4	486	60
	Reverse: 5′-GGTAAACACCCCACGAGAGGA-3′.	Exon 6		
KLK 14	Forward : 5′-CACTGCGGCCGCCCGATC-3′	Exon 3/4	485	65
	Reverse: 5′-GGCAGGGCGCAGCGCTCC-3′	Exon 6		
GADPH	Forward : 5′-TCGGAGTCAACGGATTTGGTCGTA-3′	Exon 3	305	65
	Reverse : 5′-AGCCTTCTCCATGGTGGTGAAGA-3′	Exon 5		

Table S2: Colorectal cancer cell lines origin. [1-3]

Cell line	Phenotype
**LoVo**	**Derived from metastatic site: left supraclavicular region**
CaCo2	Primary tumor of colon adenocarcinoma
HCT 116	Primary tumor of colon carcinoma
HT29	Primary tumor of colon adenocarcinoma
HCT-8	Primary tumor of colon adenocarcinoma
SW480	Primary tumor of colon adenocarcinoma
**SW620**	**Colon adenocarcinoma, derived from metastasis to lymph node of the same patient as SW480**
SW48	Primary tumor of colon adenocarcinoma
Colo205	Primary tumor of colon adenocarcinoma
Colo320 HSR	Primary tumor of colon adenocarcinoma
LS174T	Primary tumor of colon adenocarcinoma
WIDR	Primary tumor of colon adenocarcinoma
**T84**	**Colon carcinoma. Derived from metastasis to lung**

References

1. Berg, K. C.; Eide, P. W.; Eilertsen, I. A.; Johannessen, B.; Bruun, J.; Danielsen, S. A.; Bjørnslett, M.; Meza-Zepeda, M. A.; Eknæs, M.; Lind, G. E.; et.al. Multi-omics of 34 colorectal cancer cell lines-a resource for biomedical studies. *Mol. Cancer*, **2017**. *16*, 1–16. https://molecular-cancer.biomedcentral.com/articles/10.1186/s12943-017-0691-y#citeas
2. Yeh, J. J.; Routh, E. D.; Rubinas, T.; Peacock, J.; Martin, T. D.; Shen, X. J.; Sandler, R. S.; Kim, H. J.; Keku, T., O. and Der, C. J. KRAS/BRAF mutation status and ERK1/2 activation as biomarkers for MEK1/2 inhibitor therapy in colorectal cancer. *Mol. Cancer Ther.* ,**2009** *8*, 834–843. https://doi.org/10.1158/1535-7163.MCT-08-0972
3. Abajo, A.; Bitarte, N.; Zarate, R.; Boni, V.; Lopez, I.; Gonzalez-Huarriz, M., Rodriguez, J.; Bandres, E.and Garcia-Foncillas, J. Identification of colorectal cancer metastasis markers by an angiogenesis-related cytokine-antibody array. *World J. Gastroenterol. : WJG*, **2012** *18*, 637. https://www.wjgnet.com/1007-9327/full/v18/i7/637.htm
